# Reduction of fentanyl-induced cough by low-dose nalbuphine pretreatment during induction of general anesthesia in children: a randomized controlled trial

**DOI:** 10.3389/fped.2026.1795451

**Published:** 2026-06-10

**Authors:** Pan He, Yingying Tao, Yang Shen, Zhezhe Peng, Mazhong Zhang, Ying Sun

**Affiliations:** Department of Anesthesiology, Shanghai Children's Medical Center, Shanghai Jiao Tong University School of Medicine, Shanghai, China

**Keywords:** children, cough, fentanyl, low-dose, nalbuphine

## Abstract

**Background:**

Fentanyl-induced cough (FIC) during induction of general anesthesia is a common phenomenon and may cause adverse events. This study was designed to assess the efficacy and safety of low-dose nalbuphine pretreatment for the reduction of FIC in children.

**Methods:**

One hundred patients aged 2–12 years, with American Society of Anesthesiologists (ASA) physical status I–II, scheduled for elective surgery under general anesthesia, were randomly allocated to the nalbuphine group (Group N) or the control group (Group C). Patients were assigned to receive either nalbuphine 0.02 mg/kg or an equal volume of 0.9% normal saline 2 min before fentanyl bolus (2 μg/kg, injected within 5 s). The primary outcome was the incidence of FIC during induction of general anesthesia. The secondary outcomes included the onset time and severity of cough. Vital signs and any adverse events were recorded during induction of general anesthesia.

**Results:**

The incidence of FIC was significantly lower in Group N than in Group C (9/50 [18%] vs. 27/50 [54%]; risk difference, −36% (95% CI, −53% to −19%); *P* < 0.001). There was no significant difference between the two groups in the onset time of cough (Mann–Whitney *U* = 101.5, *P* = 0.460; *r* = 0.165, 95% CI, −0.173–0.468). Cough severity in Group N was statistically lower than that of Group C (Mann–Whitney *U* = 750, *P* < 0.001; *r* = 0.402, 95% CI, 0.223–0.554). No significant differences were observed in heart rate (HR), non-invasive blood pressure (NIBP) and oxygen saturation (SpO_2_) between the two groups before or after fentanyl administration. No adverse events occurred during induction of general anesthesia in either group.

**Conclusion:**

Low-dose nalbuphine 0.02 mg/kg significantly reduces the incidence of FIC during induction of general anesthesia in children.

## 1 Introduction

Fentanyl is one of the most commonly used agents for induction of general anesthesia because of its rapid onset, short duration of action, potent analgesic effect, high cardiovascular stability, and low histamine release (1, 2). However, intravenous administration of fentanyl often elicits cough (3–5). The reported incidence of fentanyl-induced cough (FIC) varies widely, ranging from 18% to 68% (6). In children, the incidence of FIC ranges from 46.3% to 60.0%, and its severity is dose-dependent (4). Although FIC is transient and clinically benign in most cases (6), occasional severe or explosive episodes still occur in children (3, 4). FIC may cause temporary increases in intraocular, intracranial, and intra-abdominal pressures (7), whereas life-threatening complications such as airway obstruction and aspiration pneumonia are extremely rare (6). It has been reported that explosive cough in children after peripheral IV injection of fentanyl can be severe enough to produce periorbital petechiae, which can be relieved only after induction of general anesthesia (3). Therefore, reducing the incidence of FIC in children is clinically relevant for perioperative comfort and safety.

Recent studies have revealed that pretreatment with low-dose opioid receptor agonist-antagonist, such as dezocine and pentazocine, can effectively suppress FIC with almost no adverse events (8–10). Nalbuphine is a semisynthetic opioid analgesic, acting as a partial *μ*-opioid receptor antagonist and a *κ*-opioid receptor agonist, and its pharmacological properties are similar to those of the above drugs. However, previous study on the antitussive effect of nalbuphine has been limited to adults and focused on sufentanil-induced cough (SIC), while its effect on FIC is still unknown. To our knowledge, this is the first randomized controlled trial to investigate the effect of nalbuphine on FIC in children.

Furthermore, the nalbuphine dose used in this prior adult study (0.3 mg/kg) is close to the recommended pediatric analgesic dose, which may increase the risk of adverse reactions. However, a low-dose regimen specifically tailored to reduce the incidence of FIC in pediatric populations has not been investigated. Given the distinct pharmacokinetic and pharmacodynamic profiles of children and the inherent differences between FIC and SIC, we conducted this study to evaluate the efficacy and safety of low-dose nalbuphine pretreatment for reducing the incidence of FIC during induction of general anesthesia in children.

## 2 Materials and methods

This prospective, randomized, double-blind, placebo-controlled trial was conducted to assess the efficacy and safety of low-dose nalbuphine pretreatment for reducing the incidence of FIC during induction of general anesthesia in children. This study was carried out between April and May 2024. Ethical approval was obtained from the Institutional Review Board of Shanghai Children's Medical Center (no: SCMCIRB-K2024066-1). The trial was registered in the Chinese Clinical Trial Registry (ChiCTR2400082986). Written informed consent was obtained from legal guardians of eligible patients for each subject before surgery. All procedures were performed in accordance with the Declaration of Helsinki.

### 2.1 Participants

A total of 105 pediatric patients aged 2–12 years, with American Society of Anesthesiologists (ASA) physical status I–II, scheduled for elective surgical procedures under general anesthesia were screened. Exclusion criteria included emergency surgery, uncooperative patients, body weight > 20% above ideal body weight, chronic cough, pre-existing respiratory diseases, a history of asthma, current use of medications that may interfere with this study (such as bronchodilators, steroids or angiotensin-converting enzyme inhibitors), refusal by parents or guardians to participate.

### 2.2 Randomization and blinding

A computer-generated random sequence was used to allocate eligible patients in a 1:1 ratio to either the nalbuphine group (Group N) or the saline control group (Group C). To ensure allocation concealment, the assignment for each participant was sealed in sequentially numbered, opaque envelopes. These envelopes were prepared and held by an independent research assistant who was not involved in subsequent patient care or outcome assessment.

Based on the allocation, the same assistant prepared identical syringes containing either nalbuphine (0.02 mg/kg, diluted to 1 mg/mL) or an equal volume of 0.9% normal saline. All syringes were labelled only with the study identification number.

Two minutes before induction of general anesthesia, the attending anesthesiologist, who was blinded to group allocation, received the corresponding syringe and administered its contents intravenously. Consequently, the participants, their guardians, the attending anesthesiologist, and the outcome assessors were all blinded to the treatment assignment throughout the trial. The randomization code was kept by the independent assistant and was not revealed until after database lock and the completion of preliminary statistical analysis. Data analysts were also kept blinded to group allocation during the initial analysis phase.

### 2.3 Anesthesia protocol and research procedure

No premedication was provided. Thirty minutes before the child entered the operating room, tetracaine hydrochloride gel was applied to the dorsum of the hand. Upon arrival in the operating room, a 24-gauge venous cannula was inserted. All injections were administered through this cannula using a dedicated infusion set without extension tubing. No other intravenous fluids were given concurrently through the same venous cannula during study drug injection. All patients were routinely monitored with electrocardiogram (ECG), heart rate (HR), non-invasive blood pressure (NIBP) and oxygen saturation (SpO_2_) throughout the study period.

Two minutes prior to fentanyl (Yichang Humanwell Pharmaceutical Co., Hubei, China) bolus, patients received either nalbuphine (Yichang Humanwell Pharmaceutical Co., Hubei, China) 0.02 mg/kg IV or equal volume of 0.9% normal saline over 5 s. The injection volume of nalbuphine was 0.02 mL/kg at a fixed drug concentration of 1 mg/mL, with the control group receiving an equal volume of normal saline in identical syringes. Following nalbuphine or saline injection, the venous cannula was flushed with 2 mL of 0.9% normal saline to standardize dead-space clearance and ensure complete delivery. Patients were then administered fentanyl 2 μg/kg (diluted with normal saline to a concentration of 10 μg/mL) within 5 s. After fentanyl injection, the onset time, frequency, and severity of FIC were recorded. Assisted mask ventilation with oxygen was applied if SpO_2_ < 95%. After a 2-minute observation period, all patients received intravenous midazolam (Nhwa Pharmaceutical Co., Jiangsu, China) 0.1 mg/kg, propofol (Fresenius Kabi Pharmaceutical Co., Beijing, China) 2–3 mg/kg, and rocuronium (HameIn Pharmaceutical Co., Germany) 0.6 mg/kg. Endotracheal intubation was performed after adequate muscle relaxation was achieved. Systolic blood pressure (SBP), diastolic blood pressure (DBP), HR, and SpO_2_ were recorded before the administration of nalbuphine or normal saline (T0), 2  minutes (T1) after nalbuphine or normal saline injection, and 2  minutes (T2) after fentanyl injection. Adverse events that occurred during induction of general anesthesia in both groups were also recorded.

### 2.4 Outcome measures

The primary outcome was the incidence of FIC, defined as the presence of at least one cough episode within 2 min after fentanyl administration. The secondary outcomes were the onset time of cough and cough severity. The analysis of cough severity was prespecified in the study protocol. The onset time was defined as the interval from the completion of fentanyl injection to the occurrence of the first cough. Cough severity was assessed using a previously validated grading system (10), which classified coughing episodes as mild (1–2 episodes), moderate (3–5 episodes), or severe (> 5 episodes), based on the total number of coughs observed within 2 min.

### 2.5 Statistical analysis

The sample size calculation was based on the primary outcome (FIC incidence) using a two-sample comparison of proportions method. A previous study reported a 60% FIC incidence in children (4). We hypothesized a 50% relative reduction in FIC incidence with nalbuphine administration. With a two-sided *α* of 0.05% and 80% statistical power, 40 patients per group were initially required. A 20% dropout rate was incorporated to set the final sample size at 50 patients per group.

Continuous data are presented as mean ± standard deviation or median (interquartile range). Categorical data are presented as numbers and percentages. Normality of the data was assessed using the Shapiro–Wilk test. The independent-samples Student's *t*-test was used to compare differences between groups for normally distributed continuous data. Homogeneity of variances was evaluated with Levene's test. The Mann–Whitney *U* test was used for between-group comparisons of non-normally distributed continuous data. Cough incidence was compared using the *χ*^2^ or Fisher's exact test. The onset time of cough and cough severity were compared using the Mann–Whitney *U* test. Serial measurements of vital signs were analyzed using repeated-measures analysis of variance (ANOVA). The assumptions of normality of residuals and homogeneity of variances were examined. The assumption of sphericity was assessed using Mauchly's test, and the Greenhouse-Geisser correction was applied when violations occurred. All analyses were performed using IBM SPSS Statistics (version 25.0, IBM Corporation, Armonk, NY, USA). A two-sided *P* value <0.05 was considered statistically significant.

## 3 Results

**Figure 1:**
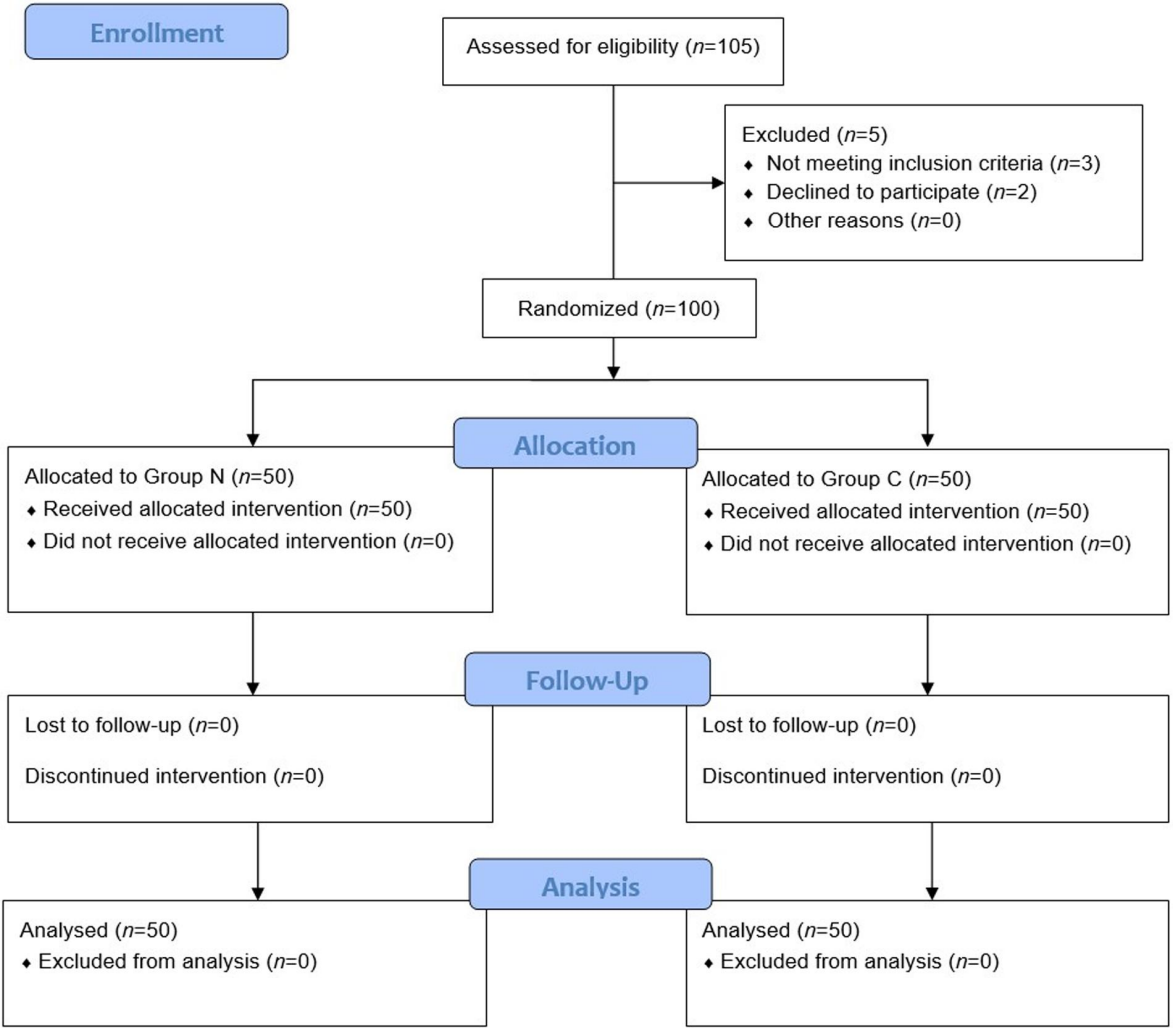
The CONSORT diagram.

Between April and May 2024, 105 pediatric patients were assessed for eligibility. Of these, 100 were enrolled and randomly assigned to either Group N (*n* = 50) or Group C (*n* = 50). No participants were lost to follow-up or discontinued the intervention. All randomized participants completed the study and were analyzed according to the intention-to-treat principle, with no missing data observed (Figure 1). Baseline characteristics of patients included in the study are summarized in Table 1. There were no statistically significant differences between the two groups in baseline characteristics.

**Table 1:** Demographic and baseline characteristics.

Characteristic	Group N (*n* = 50)	Group C (*n* = 50)	*P* value
Age (years)	6.6 ± 1.9	6.5 ± 2.0	0.887
Gender (M/F)	26/24	27/23	0.841
Weight (kg)	27.0 ± 7.9	26.1 ± 7.0	0.533
Height (cm)	123.6 ± 13.8	123.0 ± 14.3	0.823
ASA score (I/II)	28/22	26/24	0.688

ASA, American Society of Anesthesiologists Physical Status Classification. Data are presented as mean ± standard deviation or the number of patients.

No cough was observed after intravenous bolus of nalbuphine or normal saline in this study. After the fentanyl injection, the incidence of cough was significantly lower in Group N than in Group C (9/50 [18%] vs. 27/50 [54%]; risk difference, −36% [95% CI, −53% to −19%]; *χ*^2^ = 14.063, *P* < 0.001). Among patients who experienced cough, the median onset time was 6.0 s (IQR 4.5–8.0) in Group N and 5.0 s (IQR 3.0–8.0) in Group C. No statistically significant difference in cough onset time was observed between the two groups, as assessed by the Mann–Whitney *U* test (Mann–Whitney *U* = 101.5, *P* = 0.460; *r* = 0.165, 95% CI, −0.173–0.468). Cough severity was significantly lower in Group N than in Group C among all randomized participants (Mann–Whitney *U* = 750, *P* < 0.001; *r* = 0.402, 95% CI, 0.223–0.554). The incidence, onset time, and severity of cough between the two groups during the study period are shown in Table 2.

**Table 2:** Comparison of the incidence, onset time and severity of cough between the two groups.

Outcomes	Group N (*n* = 50)	Group C (*n* = 50)	*P* value
Incidence of cough (n, %)	9 (18%)	27 (54%)	<0.001*
Onset (s)	6.0 (4.5–8.0)	5.0 (3.0–8.0)	0.460
Severity of cough (n, %)			<0.001*
None	41 (82%)	23 (46%)	
Mild	5 (10.0%)	6 (12.0%)	
Moderate	3 (6.0%)	11 (22.0%)	
Severe	1 (2.0%)	10 (20.0%)	

Data are presented as number (percentage) or median (interquartile range).

* *P* < 0.05 compared with Group C.

No significant differences were observed in SBP, DBP, HR, and SpO_2_ between the two groups before or after fentanyl administration (Table 3). None of the patients suffered from apnea, hypoxemia, hemodynamic instability, nausea and vomiting, or other adverse effects during the study in either group.

**Table 3:** Changes in vital signs between the two groups at each time point.

Variables	Group N (*n* = 50)	Group C (*n* = 50)	*P* value
T0			
SBP	115.50 ± 14.455	114.68 ± 12.572	0.763
DBP	66.46 ± 10.122	66.54 ± 10.488	0.969
HR	100.26 ± 17.336	102.36 ± 18.634	0.561
SpO_2_	99.5 (99,100)	100 (99,100)	0.239
T1			
SBP	110.26 ± 13.265	112.32 ± 11.347	0.406
DBP	64.20 ± 10.587	64.82 ± 10.484	0.769
HR	97.50 ± 16.275	100.30 ± 17.696	0.412
SpO_2_	100 (99,100)	100 (99.75,100)	0.675
T2			
SBP	109.64 ± 13.320	110.80 ± 11.350	0.640
DBP	64.34 ± 10.678	63.86 ± 10.486	0.821
HR	93.72 ± 17.607	96.34 ± 17.650	0.459
SpO_2_	100 (99,100)	100 (100,100)	0.089

SBP, systolic blood pressure; DBP, diastolic blood pressure; HR, heart rate; SpO_2_, oxygen saturation; T0, time before the administration of nalbuphine or normal saline; T1, 2 min after nalbuphine or normal saline injection; T2, 2  min after fentanyl injection. Data are presented as mean ± standard deviation or median (interquartile range).

## 4 Discussion

In this prospective, randomized controlled study, we found that preemptive administration of nalbuphine 0.02 mg/kg 2 min before fentanyl bolus was associated with reduced FIC incidence without observed short-term adverse events in this pediatric cohort during induction of general anesthesia.

Fentanyl is commonly used for general anesthesia, but reflex cough, which is self-limiting and dose-dependent, frequently occurs after intravenous administration. Previous studies have demonstrated that the incidence of FIC is inversely related to age, and this may be related to the higher activity of irritant receptors in the younger population (4, 11–13). The incidence of FIC in the control group in this study was significantly higher than reported in previous adult studies (7, 10), which is consistent with an age-related trend. The patients included in our study were aged 2–12 years, a population in which young age is a known risk factor for FIC (14), which is consistent with the age-related trend described above. Similar to Han et al. (4) and Golmohammadi et al. (13), we found that administration of 2 μg/kg fentanyl induced cough reflex in 54% of children in the control group. The incidence of FIC in our control group was higher than that (43.4%) reported by Gecaj-Gashi et al. (15). This discrepancy may be explained by differences in patient age, fentanyl injection speed, or fentanyl dose between the two studies. The cough onset time of FIC in our study was shorter than that reported by Han et al. (4). This discrepancy may be related to differences in fentanyl injection speed.

Many studies have demonstrated that the incidence of FIC is dose-dependent (16–18). Currently, high-dose fentanyl and its analogs are widely used in cardiovascular surgery due to their ability to maintain hemodynamic stability and attenuate the hemodynamic response from surgical stress. These properties are particularly important in patients with severe cardiac disease (19). However, cough elicited by fentanyl is undesirable. Explosive cough can cause serious complications in patients with pre-existing cardiac dysfunction or severe coronary artery disease. Consistent with a previous study (18), we have observed a higher incidence of FIC in children undergoing cardiac surgery in our daily clinical practice. Therefore, it is of great practical significance to take effective interventions to reduce the incidence of FIC in children, especially those at high risk of FIC.

The exact mechanism of FIC remains unclear, and several hypotheses have been proposed. One proposed mechanism is the receptor hypothesis. Other hypotheses include the vagal excitation hypothesis, the *β*-arrestin signaling pathway, citrate stimulation, histamine release, neuropeptides release, and muscle stiffness (14, 20). To date, many pharmacological interventions have been used clinically to reduce the incidence of FIC in adults and children. Suppression of cough by intravenously administered lidocaine supports the peripheral cough receptors theory (15, 21, 22). Effective suppression of cough by dexamethasone might occur via the inhibition of tachykinin-mediated airway hyperreactivity (23). Dexmedetomidine can reverse muscle stiffness or relieve histamine-induced tracheal smooth muscle contraction (24). Ketamine may suppress FIC via its antagonistic effect on the N-methyl-D-aspartic (NMDA) receptor (25, 26). Although the above-mentioned medicines can partially reduce the incidence and severity of FIC, some unexpected adverse events may occur, such as arrhythmia, bradycardia, hypertension or hypotension, and allergic reactions. In recent years, studies have found that pretreatment with low-dose opioid receptor agonist-antagonist, such as dezocine and pentazocine, can effectively reduce the incidence of FIC with few adverse events (8–10). Nalbuphine is a semi-synthetic opioid receptor agonist-antagonist that acts as a partial μ-opioid receptor antagonist and a *κ*-opioid receptor agonist. Its half-life and duration of action are longer than those of other opioid agonist-antagonists, such as butorphanol and dezocine. It has the advantages of strong analgesic effect and mild adverse reactions, making it a promising option for clinical application. However, the effect of nalbuphine on FIC is still unknown. To date, relatively little research has been reported about FIC in children, and previous evidence was limited to a single adult study of SIC at a dose of 0.3 mg/kg. To our knowledge, this is the first randomized controlled trial to investigate the effect of nalbuphine on FIC in children, which is the main novelty of our study.

In our study, we found that children in Group N had a reduced incidence of cough compared with those in Group C. Potential explanations for the decreased incidence of cough are proposed as follows: First, we hypothesize that as a partial μ-opioid receptor antagonist, nalbuphine may reduce the incidence of cough through competitive inhibition. When administered before fentanyl, it may compete with fentanyl for μ-opioid receptors involved in the cough reflex, occupying these sites and thereby inhibiting fentanyl from binding and activating the receptors, potentially elevating the cough threshold. Second, in its role as a *κ*-opioid receptor agonist, nalbuphine may exert a direct central antitussive effect. Activation of *κ*-opioid receptors within brainstem cough centers may suppress the central cough reflex arc, a plausible mechanism underlying cough suppression. This central action, combined with the peripheral μ-opioid receptor antagonism described above, may reduce the excitability of vagal C-fibers. Therefore, we propose that nalbuphine may reduce the incidence of FIC through a coordinated mechanism involving both central and peripheral components of the cough reflex.

There may be special concern that pretreatment with low-dose opioid receptor antagonists prior to anesthesia may affect the analgesic effects of opioids. In fact, opioid receptors exhibit bidirectional signaling, namely excitatory (Gs) and inhibitory (Go/Gi) pathways. It is hypothesized that high-dose opioid receptor antagonists might displace opioids from Go/Gi protein-coupled receptors and may exert their antagonistic effect by blocking the analgesic effect mediated by these receptors. In contrast, low-dose antagonists may potentially displace opioids from the Gs-coupled receptors and might block hyperalgesia induced by Gs-coupled receptors, thus potentially enhancing the analgesic effect (27). A large number of studies have shown that low-dose opioid receptor antagonists may increase the analgesic effect of opioids and reduce the occurrence of adverse reactions (28–32). Therefore, pretreatment with low-dose opioid receptor antagonist to reduce FIC may potentially have certain advantages in improving the quality of induction of general anesthesia and recovery. We propose that the attenuated analgesic effect at the μ-opioid receptor might be compensated by the increased analgesic effect at the *κ*-opioid receptor. Therefore, low-dose nalbuphine may potentially enhance the analgesic effect of fentanyl. In fact, there is evidence that nalbuphine does not antagonize an opioid analgesic administered just before, concurrently, or just after an injection (33–35).

Compared with non-pharmacological measures (slower injection, dilution, site selection for venous access, priming doses), pretreatment with nalbuphine provides consistent efficacy independent of technical factors or patient cooperation. It is particularly suitable for pediatric surgeries involving intense nociceptive stimulation, for children who are unable to cooperate, and for settings requiring rapid induction. Nalbuphine may also provide preemptive analgesia and reduce perioperative opioid consumption.

In previous studies, low-dose strategies using opioid receptor agonist-antagonists have proven effective for cough suppression. For instance, Yin et al. demonstrated that butorphanol could suppress SIC at one-tenth of its conventional dose during induction of general anesthesia (36). Similarly, Xu et al. reported that intravenous dezocine could effectively reduce FIC at one-fourth of its conventional analgesic dose (9). Consistent with this low-dose antitussive pattern, we selected a nalbuphine dose of 0.02 mg/kg (approximately one-tenth to one-fifth of its pediatric analgesic dose). This dosing regimen is further supported by pediatric pharmacological considerations. First, the conventional intravenous analgesic dose of nalbuphine for children aged 2–12 years is 0.1–0.2 mg/kg (33, 34), so the present dose is well below the conventional range. Second, nalbuphine has a shorter elimination half-life and higher systemic clearance in children than in adults (37), which reduces the risk of drug accumulation at this low dose. Our dose selection was further supported by preliminary experiments, in which both 0.05 mg/kg and 0.1 mg/kg nalbuphine achieved complete suppression of FIC. These results prompted us to investigate the lower end of the effective dose range—a critical consideration for pediatric patients, as it minimizes opioid exposure.

In children, even a small additional opioid dose raises concerns about respiratory depression, sedation, and hemodynamic changes. However, our low-dose, single-injection regimen did not cause serious adverse events that required clinical intervention. Nalbuphine has a well-documented ceiling effect on respiratory depression. Although FIC in children is often transient and self-limiting, it can acutely elevate intraocular, intracranial and intra-abdominal pressures, irritate the airway, and, in rare cases, result in severe coughing episodes. The reduction in cough incidence from 54% to 18% therefore supports a favorable risk-benefit profile at this dose. Nevertheless, FIC still occurred in 18% of patients in group N, which may be due to the low dose of nalbuphine, and this dose-response relationship is consistent with prior reports. While this finding is in line with previous findings, our study provides preliminary pediatric evidence for nalbuphine in FIC prevention. We suggest that the effect of nalbuphine on FIC may be dose-dependent, and that a higher dose of nalbuphine is needed to reduce the incidence of FIC. Taking this into account, we will further explore the effects of different doses of nalbuphine on FIC.

The findings of this study could have significant clinical implications, potentially contributing to the development of a safer and more effective protocol for managing FIC in pediatric patients undergoing general anesthesia. By effectively reducing the incidence of FIC, this prophylactic strategy enhances risk control during induction of general anesthesia. It may thereby improve hemodynamic and airway stability, reduce avoidable perioperative disturbances, and support a more consistent anesthetic course. This approach could enhance patient safety and offer a rationale for optimizing management strategies in pediatric anesthesia. These findings may also inform future updates of institutional protocols for reducing cough incidence during induction of general anesthesia in pediatric patients.

However, several limitations of this study should be acknowledged. First, we did not investigate the effects of different doses of nalbuphine on the incidence or severity of FIC. Our clinical observations suggest that nalbuphine attenuates FIC in a dose-dependent manner. Therefore, future dose-finding studies are needed to determine the optimal dose. Second, the 2-minute observation window after fentanyl administration was used to collect reliable data, and this approach does not fully reflect routine clinical anesthesia practice. In addition, this window is close to the onset time of intravenous nalbuphine, which we acknowledge as a minor limitation. Third, the single-center design, specific population (ASA I–II, aged 2–12 years, elective surgery), and relatively small sample size may limit the generalizability of our findings. Our results should not be overgeneralized to other pediatric age groups, higher-risk patients (ASA ≥ III), or emergency settings. Fourth, the study was not powered for safety assessment and did not evaluate postoperative outcomes. This limits the ability to detect safety signals and restricts a comprehensive assessment of nalbuphine's clinical value in pediatric patients. Fifth, no formal assessment of blinding integrity was performed, which is acknowledged as a methodological limitation. Nalbuphine was administered at a dose of 0.02 mg/kg, which is unlikely to produce clinically detectable sedation unblinding, and all syringes were identical in appearance. Further studies are required to address these limitations.

## 5 Conclusions

In conclusion, our study suggests that administering nalbuphine 0.02 mg/kg intravenously 2 min prior to fentanyl bolus significantly reduces the incidence of FIC during induction of general anesthesia in children, without significantly affecting HR, NIBP, or SpO_2_.

## Data Availability

The raw data supporting the conclusions of this article will be made available by the authors, without undue reservation.
